# Commentary on Serre *et al.* : Demonstrating the next era of addiction science

**DOI:** 10.1111/add.16720

**Published:** 2024-11-27

**Authors:** Bryant M. Stone, Johannes Thrul

**Affiliations:** 1 Department of Mental Health, Bloomberg School of Public Health, Johns Hopkins University, Baltimore, MD, USA; 2 Sidney Kimmel Comprehensive Cancer Center at Johns Hopkins, Baltimore, MD, USA; 3 Centre for Alcohol Policy Research, La Trobe University, Melbourne, Australia

**Keywords:** Addiction research, behavioral health, ecological momentary assessment, methodological innovation, network analysis, personalized medicine

## Abstract

Innovative real-time data collection methodologies, including ecological momentary assessments, allow researchers to apply cutting-edge analytical tools, such as network analyses—unlocking insights that reshape our understanding of substance use and behavioral addictions. Serre et al.’s study epitomizes these innovative approaches poised to advance addiction research.

Persistent challenges to accurately conceptualizing and effectively treating substance use disorders (SUDs) and behavioral addictions have motivated researchers to adopt increasingly sophisticated methodologies. Among these methods, Ecological Momentary Assessments (EMA) [1–4]—real-time data collected in individuals’ daily lives—combined with cutting-edge network analyses, such as multi-level vector autoregression models and group iterative multiple model estimation (GIMME) [5–7], offer much potential to improve our understanding of substance use and other addictive behaviors. Serre *et al.*’s [1] recent study exemplifies how these methods may reshape addiction research by capturing and modeling the dynamic, moment-to-moment fluctuations in key variables (e.g., craving and self-efficacy), producing rich and highly precise information on potential treatment targets. Combining EMA as a data collection design choice with network analyses as an analytical choice, this study highlights the value of conceptualizing addictions as a personalized, fluid process requiring individualized medicine—paving the way for the next frontier of addiction research and treatments. We note two key reasons why this study’s design and analytical choice articulate this combination’s potential and impact [8].

## 1. Broad applications

Integrating EMA with network analyses enables a deeper investigation into how internal states (e.g., mood or craving) and environmental factors (e.g., distressing events) interact over time to predict substance use and behavioral addiction outcomes [9]. Compared to more traditional analytical approaches, such as mixed models, these network analyses have the advantage that they model the joint influence of all variables in the model and allow for complex time-ordered relationships between variables to emerge [10]. By capturing and analyzing real-time fluctuations in key variables preceding and following cravings, use, and experiences of withdrawal, these methods can provide nuanced insights into relapse patterns. This approach helps to address crucial questions, such as why individuals continue using or return to use substances despite negative consequences and which factors (e.g., social cues, emotional states) trigger or mitigate use and relapse.

## 2. The future of understanding and treating addictions

EMA and network analyses have the potential to transform addiction research and treatments by offering real-time tools that capture the unique complexities of substance use and behavioral addictions for each patient [6, 7], and subsequently allow us to address them in treatment. In the study by Serre *et al.* [1], EMA captured real-time fluctuations in mood, stress and social context, then a multi-level vector autoregression model mapped dynamic contemporaneous, temporal and between-subjects interactions, identifying crucial treatment targets such as craving and self-efficacy. This combined approach revealed how these variables vary within and between individuals [6, 7], emphasizing the need for flexible, personalized interventions that providers may find helpful [8–10]. If we can integrate mHealth technologies [11, 12], these methods can inform individualized interventions at the right moments, allowing clinicians to address the seemingly unpredictable and dynamic triggers of substance use behaviors with unprecedented precision. This degree of personalization far exceeds current intervention strategies in timing and precision and could improve treatment effectiveness, including relapse prevention [13].

### OVERCOMING BARRIERS AND EMBRACING INNOVATION

Despite this transformative potential of EMA and network analyses, their adoption thus far has remained limited. Researchers and clinicians may view these methods as overly complex or impractical for routine use in clinical care—currently a valid concern, given the demands of modern medical practice [14]. However, Serre *et al.* [1] demonstrate that applications of these techniques can yield significant, actionable insights. Their study shows that we can uncover individuals’ unique vulnerabilities and the nuanced variability of their behavioral patterns, the first step to paving the way for the inevitable personalization of addiction research and treatments. The greatest challenge may not lie in the mHealth tools themselves, but in demystifying these methods and highlighting successful cases such as those of Serre *et al.* (2024) [1] to inspire broader adoption and drive funding for further innovation. Addiction research is uniquely poised to lead this evolution in data collection and analysis. In doing so, we can set a standard for other medical fields, also moving towards dynamic, precise interventions. In other words, the question is no longer whether these tools can make a difference, but whether we are prepared to fully embrace them and take the next critical step.
